# Correlation of MRI quantitative perfusion parameters with EGFR, VEGF and EGFR gene mutations in non-small cell cancer

**DOI:** 10.1038/s41598-024-55033-5

**Published:** 2024-02-23

**Authors:** Mingyue Zou, Bingqian Zhang, Lei Shi, Haijia Mao, Yanan Huang, Zhenhua Zhao

**Affiliations:** 1 Department of Radiology, The Cancer Hospital of the University of Chinese Academy of Science (Zhejiang Cancer Hospital), Hangzhou, Zhejiang China; 2 Institute of Basic Medicine and Cancer (IBMC), Chinese Academy of Sciences, Hangzhou, Zhejiang China; 3 Department of Radiology, Shaoxing People’s Hospital, Shaoxing, Zhejiang China

**Keywords:** Non-small cell lung cancer, Dynamic contrast enhanced magnetic resonance imaging, Histogram, EGFR, VEGF, Biomarkers, Oncology

## Abstract

To explore the relationship between quantitative perfusion histogram parameters of dynamic contrast enhanced magnetic resonance imaging (DCE-MRI) with the expression of tumor tissue epidermal growth factor receptor (EGFR), vascular endothelial growth factor (VEGF) and EGFR gene mutations in non-small cell lung cancer (NSCLC). A total of 44 consecutive patients with known NSCLC were recruited from March 2018 to August 2021. Histogram parameters (mean, uniformity, skewness, energy, kurtosis, entropy, percentile) of each (K^trans^, K_ep_, V_e_, V_p_, F_p_) were obtained by Omni Kinetics software. Immunohistochemistry staining was used in the detection of the expression of VEGF and EGFR protein, and the mutation of EGFR gene was detected by PCR. Corresponding statistical test was performed to compare the parameters and protein expression between squamous cell carcinoma (SCC) and adenocarcinoma (AC), as well as EGFR mutations and wild-type. Correlation analysis was used to evaluate the correlation between parameters with the expression of VEGF and EGFR protein. F_p_ (skewness, kurtosis, energy) were statistically significant between SCC and AC, and the area under the ROC curve were 0.733, 0.700 and 0.675, respectively. The expression of VEGF in AC was higher than in SCC. F_p_ (skewness, kurtosis, energy) were negatively correlated with VEGF (r =  − 0.527, − 0.428, − 0.342); K^trans^ (Q50) was positively correlated with VEGF (r = 0.32); K_ep_ (energy), K^trans^ (skewness, kurtosis) were positively correlated with EGFR (r = 0.622, r = 0.375, 0.358), some histogram parameters of K_ep_, K^trans^ (uniformity, entropy) and V_e_ (kurtosis) were negatively correlated with EGFR (r =  − 0.312 to − 0.644). Some perfusion histogram parameters were statistically significant between EGFR mutations and wild-type, they were higher in wild-type than mutated (P < 0.05). Quantitative perfusion histogram parameters of DCE-MRI have a certain value in the differential diagnosis of NSCLC, which have the potential to non-invasively evaluate the expression of cell signaling pathway-related protein.

## Introduction

Lung cancer is the leading cause of cancer mortality worldwide^1^. In China, the total number of new cases and deaths of lung cancer reached 1.46 million in a single year^2^. It is noteworthy that the proportion of non-small cell lung cancer (NSCLC) has risen to 80%^3,4^. The development of cancer is believed to be mainly caused by genetic and epigenetic variations. The formation process of NSCLC is very complex. Tumor neovascularization plays an important role in its occurrence, development, invasion and metastasis. Epidermal growth factor receptor (EGFR) and vascular endothelial growth factor (VEGF) are the most important regulatory factors. EGFR is a transmembrane glycoprotein, a member of the EGFR family of tyrosine receptor kinases, also known as ErbB or HER. Its inhibition provides a promising method for the treatment of cancer^5^. VEGF (usually referred to as VEGF-A) is secreted by tumor cells under the stimulation of hypoxia environment. This factor is one of the most important factors known to promote angiogenesis and plays a role in tumor angiogenesis^6,7^. Relevant data showed that EGFR and VEGF proteins were abnormally expressed in gastric cancer, lung cancer, head and neck SCC, liver cancer and other tumors^8,9^. In recent years, with the rapid development of cancer genomics, it has been confirmed that the occurrence and development of NSCLC are driven by key oncogenes, among which EGFR gene mutation is an important driving factor to induce and maintain lung cancer, especially lung adenocarcinoma^10^. A large number of studies have shown that epidermal growth factor receptor tyrosine kinase inhibitor (EGFR-TKI) can significantly prolong the survival period of patients with EGFR sensitive mutations in advanced NSCLC^11^. Therefore, it is very important to determine the EGFR gene mutation status of patients through tumor tissue for making personalized treatment plans for NSCLC patients. However, on the one hand, some patients cannot endure pathological detection. On the other hand, tissue samples cannot fully reflect the condition of the lesion, and the relevant parameters before and after treatment neither be dynamically observed.

The development of imaging provides a feasible detection technology for evaluating the microcirculation of NSCLC. CT scanning has the advantages of high time resolution and simple operation. However the CT features which focus on the morphology are lack of quantitative and functional analysis^12^. Dynamic Contrast Enhanced Magnetic Resonance Imaging (DCE-MRI), as a new biomarker, has been used to evaluate the anti-angiogenesis effect of various tumors, including renal cell carcinoma, pancreatic cancer and colorectal cancer with liver metastasis. It quantitatively detects tumor microvascular parameters such as K^trans^ (transfer constant), V_p_ (fractional volume of plasma), K_ep_ (rate constant of backflux from extravascular extracellular space [EES] to plasma), V_e_ (fractional volume of EES) and F_p_ (tissue plasma perfusion) were used to evaluate tumor blood perfusion and vascular wall permeability^13^. DCE-MRI quantitative perfusion parameters provide functional information of tumor angiogenesis, while histogram analysis mostly uses descriptive parameters, including mean, kurtosis, energy, skewness, entropy and percentiles, to quantitatively describe and compare the distribution of tumor biomarkers. In tumor diagnosis and treatment, it is mainly used in the differentiation of benign and malignant tumors, tumor classification and grading, response evaluation after radiotherapy or chemotherapy, and treatment effect prediction^14,15^.

In our study, DCE-MRI histogram parameters of quantitative perfusion indexes (K^trans^, K_ep_, F_p_, V_p_, V_e_) were used to explore the value in differential diagnosis of pathological types of NSCLC, and to evaluate the correlation between microvascular parameters of NSCLC with EGFR gene mutation and EGFR, VEGF protein expression, so as to provide a non-invasive and repeatable imaging detection means for the detection of the microvascular status of NSCLC in vivo.

## Materials and methods

### Patient population

This prospective study was approved by the Institutional Medical Research Ethics Committee. 70 consecutive patients were recruited who underwent lung DCE-MRI in our hospital from March 2018 to August 2021.

Inclusion criteria: (1) Those suspected to be lung malignant tumors by routine imaging examinations, and the diameter of the lesions > 2 cm; (2) Pathological and clinical data can be obtained; (3) No radiotherapy, chemotherapy and other treatments were performed before MRI examination, surgery or puncture.

Exclusion criteria: (1) Patients with other pulmonary diseases confirmed by pathological results. (2) The image scanning quality is poor or the tumor boundary can not be clearly delineated. (3) The sample tissue used for molecular pathological detection is insufficient or of poor quality.

Finally, a total of 44 NSCLC (38 male and 6 female, age range was 47–85 years) were included in the study, and all patients provided informed consent form. There were 22 patients with SCC and 22 with AC confirmed by pathological biopsy. The general characteristics of lung cancer of two pathological types were detailed in Table 1. There was no statistical difference in age and BMI between two groups (t = 0.015, P = 0.691; t = -0.763, P = 0.445).

**Table 1:** Summary of 44 clinical characteristics [n (%), ($\overline{x}$ ± s), M (P25, P75)].

	SCC (n = 22)	AC (n = 22)	t/z value	*P* value
Sex				0.188
Male	21 (95.5%)	17 (77.3%)		
Female	1 (4.5%)	5 (22.7%)		
Age (years, x ± s)	67.0 ± 9.9	65.8 ± 9.7	0.015	0.691
Range	47–85	50–84		
BMI	21.3 ± 2.7	22.0 ± 4.7	− 0.763	0.445

### Scanning method

Lung MRI was performed on a 3.0 T MR scanner (Siemens Vefio, Germany), and the scanning parameters of multiple flip angle were as follows: TR 3.25 ms, TE 1.17 ms, layer thickness 5 mm, number of layers 30, field of view 350 mm × 282 mm, flip angle: 5°, 10°, 15°, matrix 162 × 288. 3D VIBE T1 weighted dynamic perfusion sequence was applied to dynamic enhanced scanning. Subsequently, a gadolinium contrast agent (Omega, GE Healthcare, China) was injected through the median elbow vein using the high-pressure injector in phase 3. The injection speed and dose were and 3.5 ml/s and 0.1 mmol/kg, respectively. Thereafter, multi-phase dynamic enhanced scanning was performed with the following parameters: flip angle: 10°, scanning phases: 35, totaling time: 247 s; the other parameters were the same as above.

### Perfusion analysis

**Figure 1:**
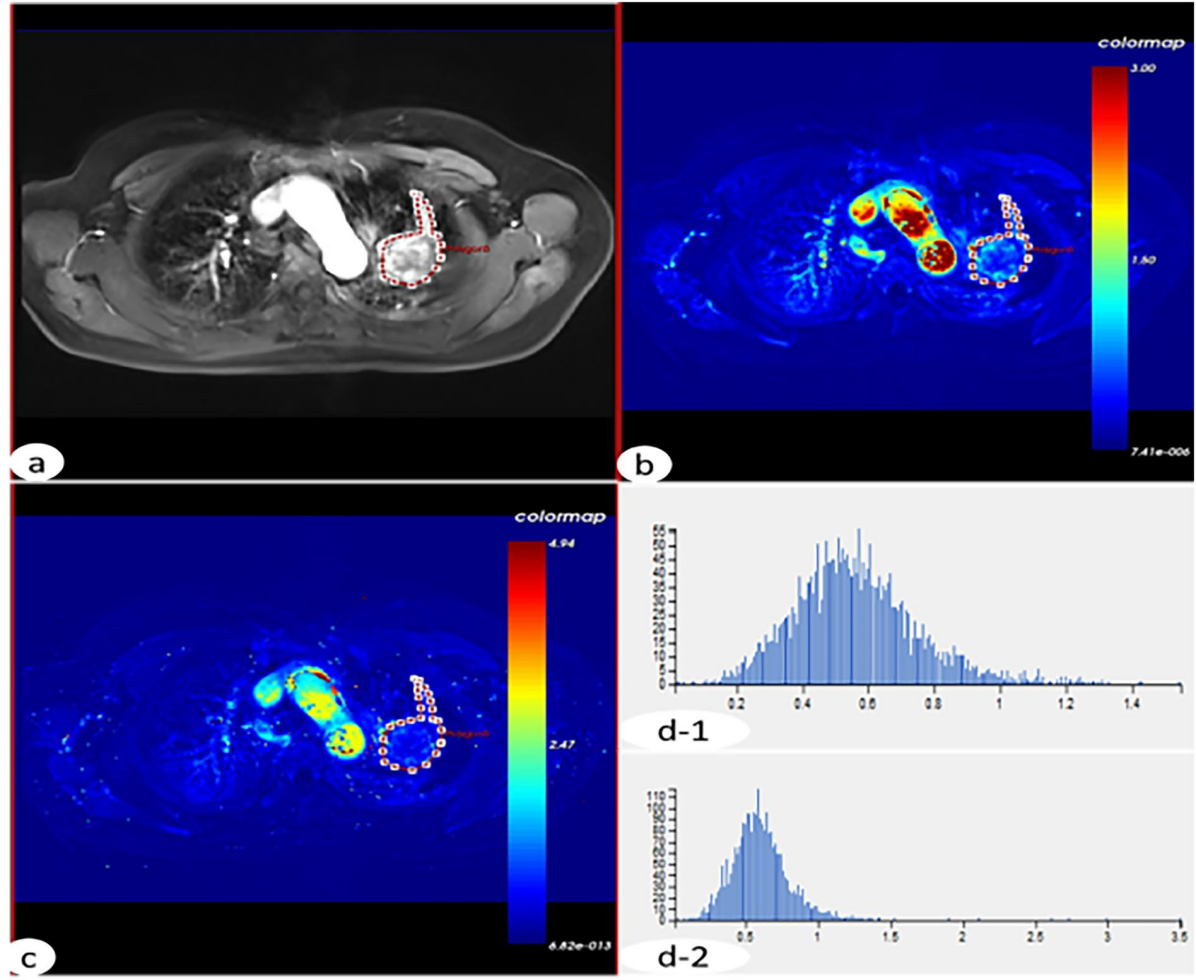
A 52-year-old female, was pathologically diagnosed as adenocarcinoma of the left upper lung. (**a**) The original DCE-MR image of the patient’s chest, and the red circle area is the focus; (**b**) the F_p_ image of the corresponding EC model, and the red circle area is the focus; (**c**) the corresponding EC model KTrans image, and the red circle area is the focus. (**d-1**) The histogram of the Fp perfusion parameters of the EC model (mean = 0.5639; homogeneity = 0.6543; skewness = 0.6992; kurtosis = 1.2016; energy = 0.0111; entropy = 6.7617). (**d-2**) The histogram of the KTrans perfusion parameters of the EC model of the lesion (mean = 0.6077; uniformity = 0.6358; skewness = 2.5321; kurtosis = 22.0993; entropy = 5. 7501; energy = 0.0229).

The different flip angles (5°, 10°, 15°) of T1 weighted and dynamic enhanced images were imported into the hemodynamic software Omni Kinetics (GE Healthcare, China). The 3D correction technology of free breathing (no rigid medical image registration algorithm) was used to correct the motion artifacts of dynamic enhancement sequence and check the fitting state of time intensity curve (TIC) before and after registration and check the registration effect. The pulmonary artery and thoracic aorta were selected as the region of interest (ROI) of the blood supply artery using dual blood supply model (UserAIF). We Selected an Exchange hemodynamic model to calculate quantitative perfusion parameters^16,17^, avoiding cystic change, necrosis and normal lung tissue as far as possible to sketch the ROI, 3–5 layers above and below the largest tumor layer were integrated into a 3D ROI for quantitative analysis and calculation, including K^trans^, K_ep_, V_e_, V_p_, and F_p_. Two experienced radiologists measured the perfusion parameters for three times respectively, and then took the average value as the final measurement value (Fig. 1).

### Immunohistochemical evaluation of VEGF and EGFR

**Figure 2:**
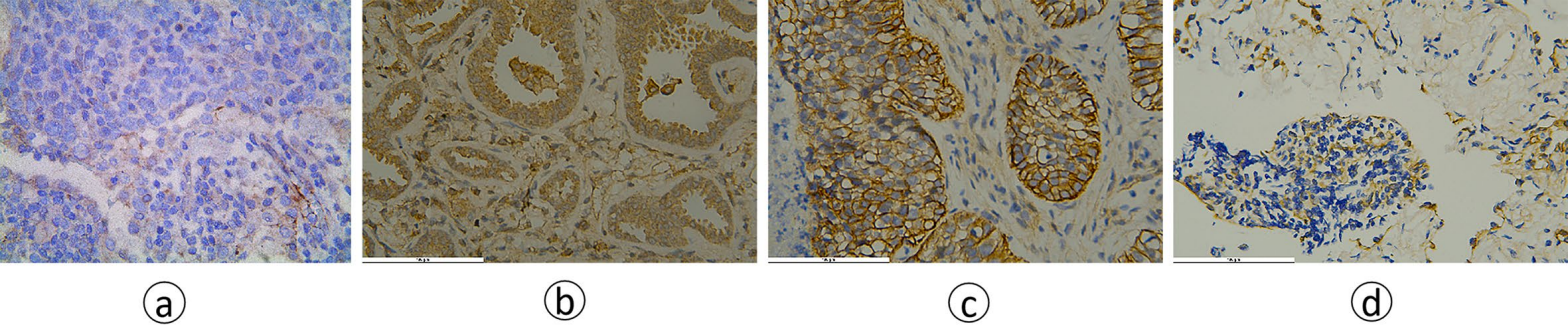
Immunohistochemical staining of different proteins for different pathological types. (**a**) VEGF, SCC, IRS:1; (**b**) VEGF, AC, IRS:4; (**c**) EGFR, SCC, IRS:9; (**d**) EGFR, AC, IRS:2 (magnification × 400).

VEGF and EGFR were assessed by immunohistochemistry (IHC) using paraffin‐embedded tissue samples which were obtained from surgery or biopsy. Briefly, all sections were deparaffinized and rehydrated, and antigen retrieval was performed before immunohistochemical staining. Non‐specific binding sites were blocked by serum blocking solution at 37 °C for 10 min (Dako company). The sections were stained with monoclonal mouse anti‐human VEGF antibody and EGFR antibody at 4 °C overnight. The specimens were stained with secondary antibody and were then incubated at 37 °C for 10 min. DBA staining, rinsing, mild counterstaining of hematoxylin. After dehydration, transparency and mounting, the slides were visualized using a microscope. The whole field of tissue was observed under a low-power microscope, and then three fields were randomly selected under high-power fields (X40) in each case. The specific calculation method of IRS was as follows: the staining intensity (SI) grading: 0, 1, 2, 3 respectively represented no color, light yellow, brown yellow, dark brown; Percentage of stained cells (PP): 0, 1, 2, 3, 4 points represented no color, stained cells < 10%, stained cells 11–50%, stained cells 51–80%, stained cells > 81%, IRS = SI × PP. Finally, take the average of three numbers (Fig. 2). Molecular analysis of mutation status of EGFR exons 18, 19, 20, and 21 was examined with a polymerase chain reaction based ARMS obtained from clinical history.

### Statistical analyses

**Figure 3:**
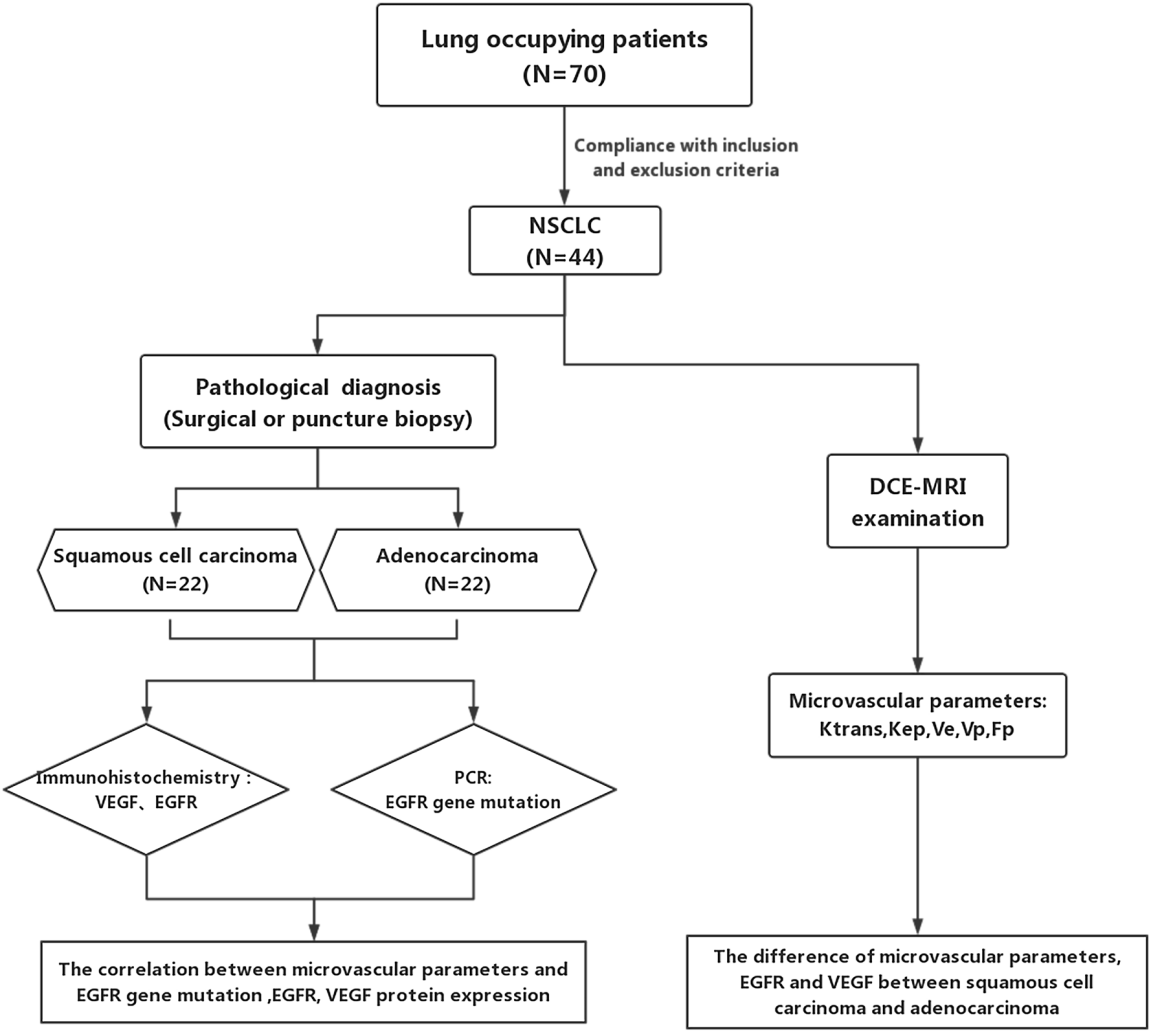
Patient flow diagram.

All data were analyzed with the statistical software IBM SPSS Statistics 25.0 and Graphpad prism. The differences in quantitative parameters and protein expression between SCC group and AC group of NSCLC were compared, and the differences in quantitative parameters between EGFR gene mutation type and wild type in NSCLC were compared, which was consistent with normal distribution using Student t test, and non normal distribution using Mann–Whitney U test. Pearson linear correlation analysis and Spearman correlation analysis were used to evaluate the correlation between microvascular parameters and related protein expression. *P* value < 0.05 was considered statistical significance (Fig. 3).

### Ethics approval

This study was performed in line with the principles of the Declaration of Helsinki. Approval was granted by the Institutional Ethics Committee of Shaoxing People’s Hospital (approval No. 2018–101).

## Results

### Perfusion histogram parameters in different pathological types of non-small cell lung cancer

**Figure 4:**
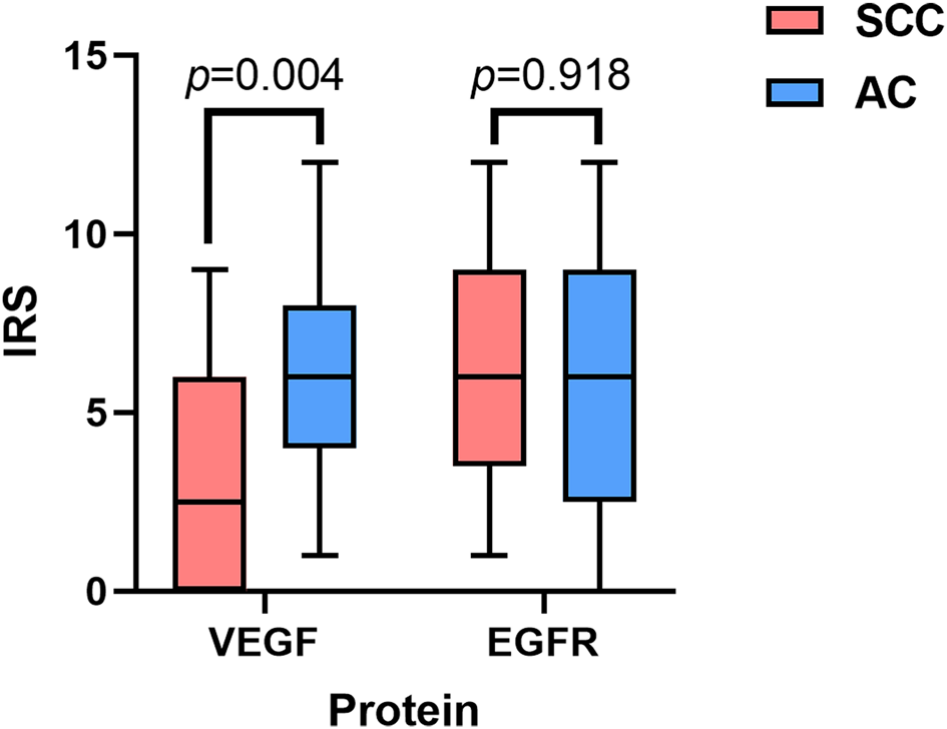
The ROC curve of F_p_ (skewness, kurtosis, energy) to identify different pathological types of NSCLC.

The results showed that there was no significant difference in (K^trans^, K_ep_, V_e_, V_p_) histogram parameters between SCC and AC (*P* > 0.05). F_p_ (skewness, kurtosis, and energy) were statistically significant between SCC and AC, and they in SCC group were higher than AC group (*P* = 0.008; *P* = 0.023; *P* = 0.047) (Table 2), and the area under the ROC curve was 0.733, 0.700, and 0.675 respectively (Fig. 4).The sensitivity of F_p_ skewness in distinguishing SCC and AC was 71.4%, the specificity is 78.3%, and cutoff value was 0.497; As for F_p_ kurtosis, sensitivity was 52.4% and specificity was 87%, when cutoff value was 0.394; The sensitivity and specificity of F_p_ energy in distinguishing SCC and AC were 66.7% and 69.6%, and cutoff value was 0.363.

**Table 2:** F_p_ perfusion histogram parameters between SCC and AC [n (%), ($\overline{x}$ ± s), M (P25, P75)].

Characteristics	SCC (n = 22)	AC (n = 22)	Z value	*P* value
Mean	0.17 (0.06, 0.35)	0.15 (0.11, 0.467)	− 0.44	0.681
Skewness	1.39 (0.82, 1.85)	0.64 (0.34, 1.01)	− 2.64	0.008*
Kurtosis	3.11 (1.01, 5.71)	0.94 (− 0.21, 0.94)	− 2.27	0.023*
Uniformity	0.45 (0.20, 0.59)	0.49 (0.42, 0.56)	− 0.83	0.404
Energy	0.01 (0.09, 0.14)	0.01 (0.01, 0.01)	− 1.99	0.047*
Entropy	6.83 (6.44, 7.08)	7.01 (6.76, 7.29)	− 1.80	0.072
Q5	0.05 (0.016, 0.12)	0.38 (0.02, 0.12)	− 0.04	0.972
Q10	0.08 (0.02, 0.16)	0.06 (0.04, 0.17)	− 1.29	0.897
Q25	0.10 (0.10, 0.43)	0.85 (0.65, 0.28)	− 0.41	0.681
Q50	0.12 (0.58, 0.33)	0.13 (0.10, 0.43)	− 0.74	0.459
Q75	0.17 (0.08, 0.44)	0.21 (0.13, 0.60)	− 0.72	0.474
Q90	0.24 (0.11, 0.56)	0.27 (0.18, 0.81)	− 0.67	0.503
Q95	0.40 (0.13, 0.64)	0.31 (0.22, 0.91)	− 0.39	0.698

With asterisks (*P < 0.05).

### Expression of EGFR and VEGF in different pathological types of NSCLC

**Figure 5:**
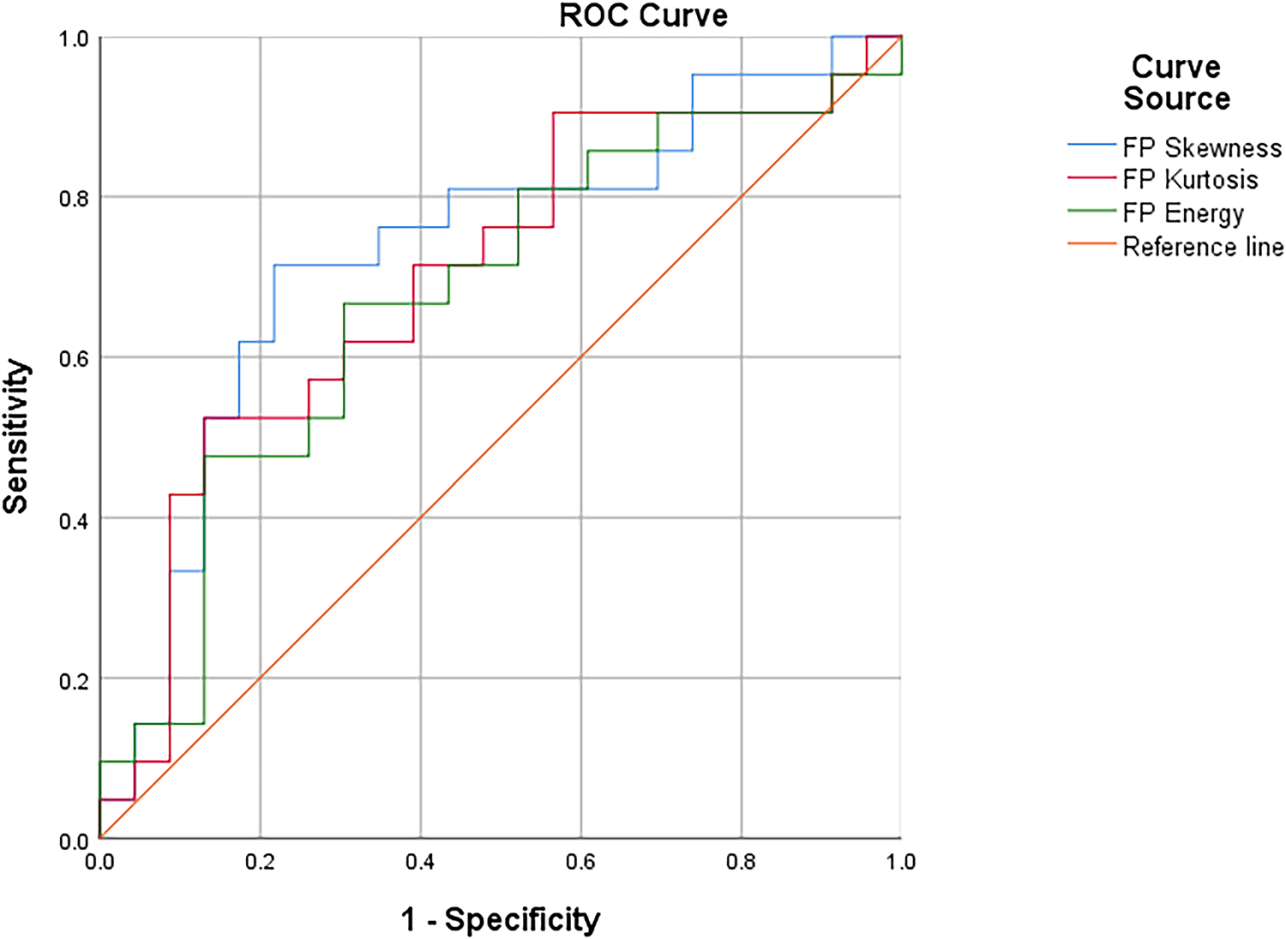
The expression of EGFR and VEGF protein in different pathological types of lung cancer. The blue represented SCC, while the red represented AC. The immunohistochemical score of VEGF was statistically significant (*P* = 0.004) in SCC; There was no significant difference in EGFR immunohistochemical score between SCC and AC (*P* = 0.918).

The expression of VEGF in SCC and AC is different, VEGF protein in AC was higher than SCC (*P* = 0.004). There was no difference in EGFR expression between SCC and AC (*P* > 0.05) (Fig. 5).

### Correlation between perfusion histogram parameters with the expression of VEGF and EGFR

**Figure 6:**
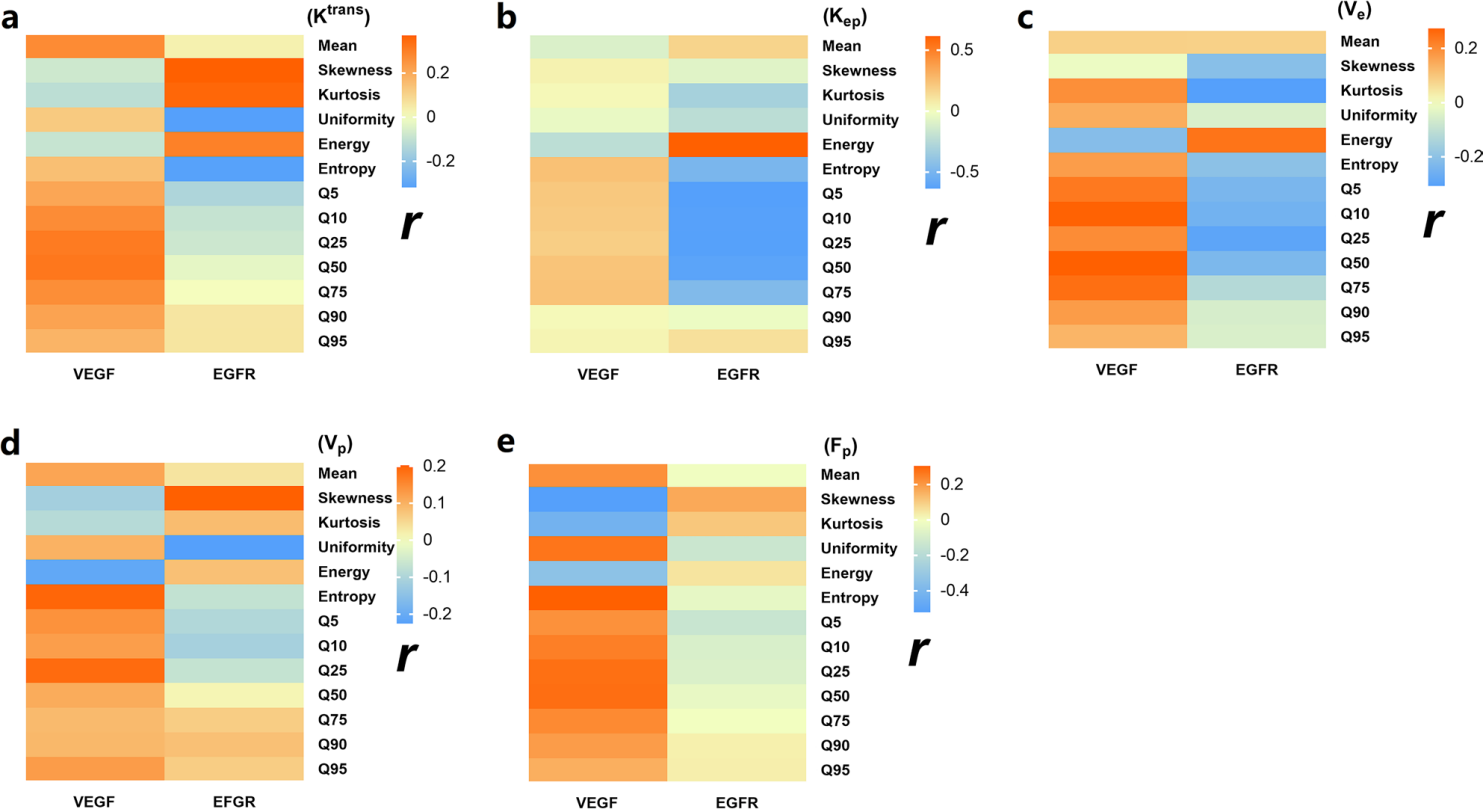
The correlations between perfusion histogram parameters with VEGF and EGFR (r). Spearman correlation analysis showed that F_p_ (skewness, kurtosis, energy) were negatively correlated with VEGF expression (r =  − 0.527, − 0.428, − 0.342; *P* < 0.001, *P* = 0.005, *P* = 0.029). K^trans^ (Q50) was positively correlated with VEGF protein expression (r = 0.32, *P* = 0.041). K_ep_ (energy) and K^trans^ (skewness and kurtosis) were positively correlated with EGFR protein expression (r = 0.622, *P* < 0.001), (r = 0.375, 0.358, *P* = 0.014, 0.02). K_ep_ (kurtosis, entropy, Q5, Q10, Q25, Q50, Q75), K^trans^ (homogeneity, entropy), V_e_ (kurtosis) were negatively correlated with EGFR expression (r =  − 0.316, − 0.498, − 0.644, − 0.638, − 0.640, − 0.623, − 0.469, *P* = 0.042, *P* = 0.001, *P* < 0.001, *P* < 0.001, *P* < 0.001, *P* < 0.001, *P* = 0.002), (r =  − 0.323, − 0.319, *P* = 0.037, 0.039), (r =  − 0.312, *P* = 0.045).

Figure 6 showed that F_p_ (skewness, kurtosis, energy) were negatively correlated with VEGF expression (*P* < 0.05); K^trans^ (Q50) was positively correlated with VEGF protein expression (*P* = 0.041). About EGFR, K_ep_ (energy) and K^trans^ (skewness and kurtosis) were positively correlated with EGFR (*P* < 0.05); K_ep_ (kurtosis, entropy, Q5, Q10, Q25, Q50, Q75), K^trans^ (homogeneity, entropy), V_e_ (kurtosis) were negatively correlated with EGFR expression (*P* < 0.05). Other parameters were not statistically significant.

### Correlation between perfusion parameters with EGFR gene expression in NSCLC

Among the 44 patients with NSCLC, 20 patients were tested for EGFR gene, including 12 EGFR gene mutations and 8 wild type. Some histogram parameters of F_p_, K_ep_ and K^trans^ were statistically significant between EGFR mutation and wild-type, and histogram parameters were all higher in the wild-type than EGFR mutation (Table 3). V_e_ and V_p_ histogram parameters were no significant difference between two groups (*P* > 0.05). The area under the F_p_ histogram parameter ROC curve were 0.771–0.792, the area under K_ep_ (entropy, Q50, Q75) ROC curve were 0.854, 0.831, 0.771, and AUC value of K^trans^ histogram parameter were (0.771–0.823). F_p_ (mean), K_ep_ (entropy) and K^trans^ (Q75) achieved high sensitivity and specificity.

**Table 3:** Perfusion histogram parameters of EGFR mutation and wild-type [n (%), ($\overline{x}$ ± s), M (P25, P75)].

Characteristics	Gene mutation (n = 12)	Gene wild-type (n = 8)	Z value	*P* value
F_p_ mean	0.12 (0.74, 1.10)	0.20 (0.14, 1.10)	− 2.16	0.031
F_p_ Q5	0.04 (0.02, 0.05)	0.06 (0.04, 0.20)	− 2.08	0.037
F_p_ Q10	0.05 (0.03, 0.06)	0.09 (0.06, 0.35)	− 2.01	0.045
F_p_ Q25	0.08 (0.05, 0.10)	0.13 (0.08, 0.69)	− 2.01	0.045
F_p_ Q50	0.11 (0.07, 0.15)	0.18 (0.13, 1.06)	− 2.08	0.037
F_p_ Q75	0.15 (0.10, 0.21)	0.26 (0.16, 1.40)	− 2.01	0.045
F_p_ Q95	0.22 (0.14, 0.34)	0.39 (0.25, 1.99)	− 2.01	0.045
K_ep_ entropy	6.96 (6.77, 7.19)	7.15 (6.58, 7.33)	− 2.62	0.009
K_ep_ Q50	0.18 (0.04, 0.25)	0.38 (0.30, 0.85)	− 2.47	0.014
K_ep_ Q75	0.53 (0.37, 0.75)	0.90 (0.81, 1.20)	− 2.01	0.045
K^trans^ mean	0.16 (0.08, 0.22)	0.29 (0.21, 1.23)	− 2.24	0.025
K^trans^ Q10	0.06 (0.04, 0.07)	0.11 (0.07, 0.38)	− 2.08	0.037
K^trans^ Q25	0.09 (0.06, 0.12)	0.18 (0.10, 0.78)	− 2.01	0.045
K^trans^ Q50	0.13 (0.08, 0.17)	0.24 (0.17, 1.21)	− 2.24	0.025
K^trans^ Q75	0.17 (0.11, 0.24)	0.32 (0.26, 1.58)	− 2.39	0.017
K^trans^ Q90	0.25 (0.14, 0.35)	0.51 (0.32, 1.91)	− 2.32	0.021
K^trans^ Q95	0.30 (0.17, 0.60)	0.70 (0.43, 2.14)	− 2.08	0.037

## Discussion

DCE-MRI is a non-invasive functional imaging technology, which can evaluate tumor vascularization by measuring the sequential change of signal intensity with time after the application of contrast agent. It is sensitive to tumor perfusion parameters such as vascular permeability, vascular density, volume and flow. It has been confirmed that it is related to tumor microvessel density (MVD), differentiation of benign and malignant, grading and classification, and evaluation of radiotherapy and chemotherapy treatment effect, which is a functional imaging technology that can reflect tumor microstructure and predict its behavior by many studies^18,19^. Several previous studies have confirmed that DCE-MRI perfusion parameters (maximum enhancement rate and K^trans^) are related to tumor angiogenesis in lung cancer animal models^20,21^. Unlike the previous studies using conventional imaging analysis, we used DCE-MRI based histogram analysis. Recently, histogram analysis has attracted much attention as a technique to evaluate the biological heterogeneity of tumors. It has reported that the histogram index is a potential tool for improving tumor differentiation, grading and treatment response evaluation^22,23^.

Some studies found that there was no significant difference in DCE-MRI quantitative perfusion parameters and VEGF between SCC and AC^24,25^. The results of our study showed that the expression of VEGF in SCC was lower than in AC, and F_p_ (skewness, kurtosis, energy) of SCC was higher than that AC. F_p_ represents intravascular plasma flow. The previous study showed that tumor progression appears to be linked to expansion of histograms to right (decreased skewness) and peak broadening with decreased height (decreased kurtosis)^26,27^. Different biological characteristics determine that AC is more prone to cellular and hematogenous metastasis than SCC. The above viewpoints were consistent with our research results. It shows that F_p_ histogram parameters and VEGF have certain value in differentiating SCC from AC.

VEGF is the strongest vascular growth factor that has been proved to induce tumor angiogenesis. VEGF and VEGF receptors (VEGFRs) play a central role in angiogenesis, promoting endothelial cell proliferation, migration and invasion^28^. F_p_ (skewness, kurtosis, energy) were negatively correlated with the expression of VEGF. Research showed that the smaller skewness and kurtosis, the higher tumor heterogeneity. The increase of tumor heterogeneity leaded to the increase of malignancy and the formation of neovascularization, which represented the increase of VEGF expression^29–31^, which was consistent with the results of our study. It is confirmed that the above parameters can indirectly evaluate the expression of VEGF protein in tumor tissue. EGFR not only participates in the differentiation and proliferation of cancer cells, promotes the apoptosis of cancer cells, but also participates in the formation of new blood vessels in cancer tissues, and is closely related to the growth and metastasis of cancer cells^32^. Our study found that K_ep_ (energy) was positively correlated with EGFR protein expression, while K_ep_ (entropy) and K^trans^ (entropy) were negatively correlated with EGFR protein expression. Entropy represents the complexity of texture in the image, which is opposite to energy. The greater energy reflected the lower internal heterogeneity of the tumor and lower malignant degree of the tumor, correspondingly, the higher expression of EGFR protein, which was in contradiction with our expectations. The results may be related to the limited number of cases and different pathological types of NSCLC. Further prospective and expanded sample size are needed to study. The results of our study showed that K_ep_ (kurtosis, Q5–Q75), K^trans^ (homogeneity) were correlated with EGFR protein expression. In studies of gastric cancer and colorectal cancer, it is inclined to think that K_ep_ and K^trans^ were significantly correlated with the expression of EGFR^33,34^, indicating that K_ep_ and K^trans^ have statistical significance in the expression of EGFR.

Our study found that F_p_ (mean, Q5–Q95), K_ep_ (entropy, Q50, Q75), and K^trans^ (mean, Q10–Q95) had statistically significant differences between EGFR wild type and gene mutation, and the wild type were higher than mutant type. The area under the curve of the above quantitative perfusion histogram parameters for identifying EGFR gene mutations were higher than 0.771, which had good diagnostic efficacy. Some studies found that in NSCLC patients, the perfusion parameters BF, BV and PS of the EGFR mutant group were higher than those of wild group, while MTT was higher in the wild group^35,36^. Yuan et al.^24^ pointed out that perfusion parameter was no significant difference between EGFR mutant group and wild-type group. There were some differences in the research results. We speculated that the tissue microstructure complexity (such as tumor cell density, tumor interstitial volume, and membrane structure complexity) of EGFR wild type may be significantly different from mutant type. The entropy value of the wild group was higher than mutant group, reflecting the high internal heterogeneity of the wild group and the high degree of malignancy of the tumor, inducing the formation of tumor blood vessels and the increase of immature blood vessels, thus leading to the increase of the mean and percentile of K^trans^ and K_ep_.

This study has several limitations: first, we have made a complete tumor measurement on DCE-MRI images, but only a small number of tumors have been histopathologically examined, which may limit our relevant results. Further prospective studies are needed to confirm our preliminary results. Secondly, this study is a single-centre sample study, and the limited sample size might have led to slight selection bias. In addition, NSCLC patients with different degrees of differentiation and pathological stages were not grouped for study, and further study will be carried out in the future.

## Conclusion

DCE-MRI quantitative perfusion histogram parameters have certain value in the differential diagnosis of NSCLC. It is feasible and reliable for evaluating the EGFR gene mutation status and the expression of VEGF and EGFR proteins in NSCLC. It may can provide a non-invasive evaluation method to predict the expression of proteins related to cell signal transduction pathway with molecular imaging parameters.

## Data Availability

The datasets generated during and/or analysed during the current study are available from the corresponding author on reasonable request.
